# DRFnet: Dynamic receptive field network for object detection and image recognition

**DOI:** 10.3389/fnbot.2022.1100697

**Published:** 2023-01-10

**Authors:** Minjie Tan, Xinyang Yuan, Binbin Liang, Songchen Han

**Affiliations:** School of Aeronautics and Astronautics, Sichuan University, Chengdu, China

**Keywords:** receptive field, neural network, object detection, image recognition, biologically inspired vision

## Abstract

Biological experiments discovered that the receptive field of neurons in the primary visual cortex of an animal's visual system is dynamic and capable of being altered by the sensory context. However, in a typical convolution neural network (CNN), a unit's response only comes from a fixed receptive field, which is generally determined by the preset kernel size in each layer. In this work, we simulate the dynamic receptive field mechanism in the biological visual system (BVS) for application in object detection and image recognition. We proposed a Dynamic Receptive Field module (DRF), which can realize the global information-guided responses under the premise of a slight increase in parameters and computational cost. Specifically, we design a transformer-style DRF module, which defines the correlation coefficient between two feature points by their relative distance. For an input feature map, we first divide the relative distance corresponding to different receptive field regions between the target feature point and its surrounding feature points into N different discrete levels. Then, a vector containing N different weights is automatically learned from the dataset and assigned to each feature point, according to the calculated discrete level that this feature point belongs. In this way, we achieve a correlation matrix primarily measuring the relationship between the target feature point and its surrounding feature points. The DRF-processed responses of each feature point are computed by multiplying its corresponding correlation matrix with the input feature map, which computationally equals to accomplish a weighted sum of all feature points exploiting the global and long-range information as the weight. Finally, by superimposing the local responses calculated by a traditional convolution layer with DRF responses, our proposed approach can integrate the rich context information among neighbors and the long-range dependencies of background into the feature maps. With the proposed DRF module, we achieved significant performance improvement on four benchmark datasets for both tasks of object detection and image recognition. Furthermore, we also proposed a new matching strategy that can improve the detection results of small targets compared with the traditional IOU-max matching strategy.

## 1. Introduction

A large number of biological experiments discovered that the receptive field (RF) of the primary visual cortex of the visual system is dynamic and can be modified by the sensory environment (Hubel and Wiesel, 1962; Cavanaugh et al., 2002; Angelucci et al., 2017). A neuron can be activated by a simple stimulus (e.g., a light spot within RF), but its response can also be modulated by a stimulus located outside RF, meaning that the neuron's response to local image properties is influenced by the context in which the local feature is embedded. The well-known center-surround RF theory (Series et al., 2003) shows the rather complicated but intelligent behavior of neurons in the primary visual cortex, revealing that the dynamic response arises from co-stimulation of CRF (classical receptive field) excitation and peripheral nCRF (non-classical receptive field) inhibition. These show that the receptive field and its corresponding information-processing mechanisms are dynamic in a biological visual system. However, in most CNN networks, the responses of a unit usually come from a kernel (e.g., equal to a receptive field of a visual neuron) with a fixed size (but also see Dai et al., 2017), where a deformable convolutional network is developed. This is a simple simulation of visual neurons but not enough to capture the dynamic properties of a real neuron and possess the capability of processing the hierarchical and comprehensive information.

In general, traditional CNN-based object detection models only capture local information within one layer (Simonyan and Zisserman, 2014; Szegedy et al., 2015; He et al., 2016). Intuitively, a large receptive field is more beneficial for object detection and instance segmentation tasks because the comprehensive information from distant neighborhoods can be used to learn the relationship between the object and the context (Hu H. et al., 2018). Nevertheless, directly increasing the kernel size will greatly increase the computational complexity and memory footprint. Therefore, the remote dependencies between object and context are generally captured by repeated local operations such as increasing the network depth, which may also introduce other issues, such as gradient disappearance and optimization difficulties (Nielsen, 2015; He et al., 2016).

The inception network (Szegedy et al., 2015) attempts to achieve diverse information from a larger and adaptive RF by juxtaposing branches of different kernel sizes and increasing the network width at the cost of huge computing costs. SKNet (Li et al., 2019) provides an earlier attempt to imitate the mechanism of adaptive RF, but its implementation uses parallel convolution kernels of different sizes to generate multiple feature maps, and then uses an attention structure to learn the channel weights of different feature maps generated from kernels of different sizes. It just fuses information from a very limited number of branches (e.g., 2 or 3). The method based on the self-attention mechanism (Vaswani et al., 2017; Wang et al., 2018; Srinivas et al., 2021) considers all the global information when calculating the excitation output of a spatial position, which can be considered to greatly increase the size of RF. However, the memory and computational cost are quadratic with the dimension of the feature map, which is very challenging for training in some computer vision tasks, such as object detection.

**Figure 1:**
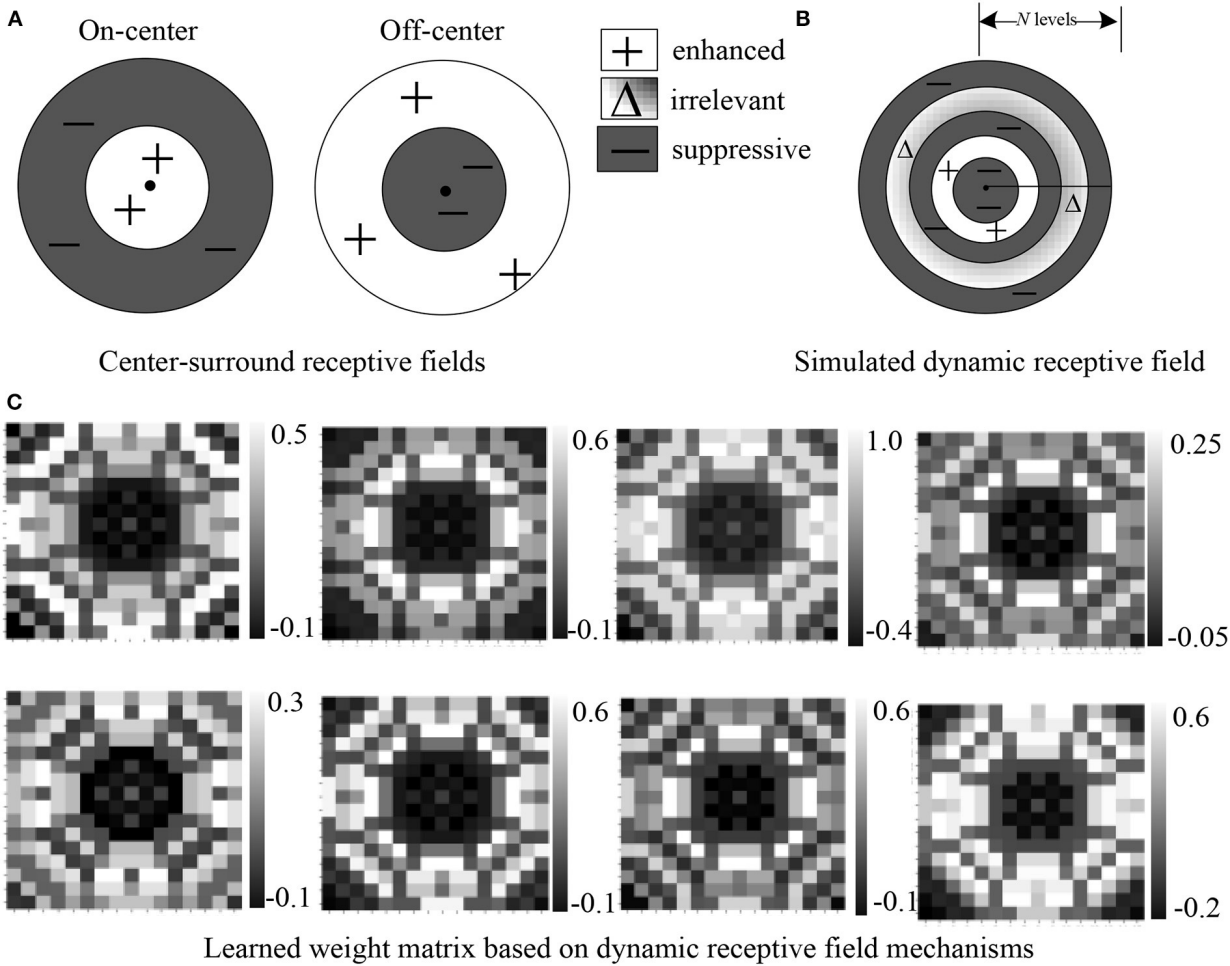
Our proposed dynamic receptive field module **(B)** imitates the biological mechanisms of center-surround receptive fields of both On-center and Off-center neurons **(A)**. A simulated dynamic receptive field can be divided into various regions of *N* discrete levels where each region is assigned a weight indicating enhancement, suppression, or no change. **(C)** Visualization of eight weight maps with positive and negative values learned from the dataset. We can see that the automatically learned weight matrix based on DRF is quite similar to **(B)**.

Our motivation is to develop a method to simulate the dynamic RF mechanism of visual neurons as shown in Figure 1, which can adaptively generate excitatory or inhibitory responses to different receptive fields in one operation module. Our model mainly simulates the dynamic receptive field mechanisms of neurons in V1 due to the rich studies of center-surround interaction properties in V1 neurons according to various stimulus conditions, which are considered to be very important for context information processing of vision (Cavanaugh et al., 2002; Angelucci et al., 2017). Inspired by the center-surround RF theory (Series et al., 2003) that points out the RF mainly consists of a central region and a surrounding region (Figure 1A), we abstract the RF of a unit in the neural network into a shape made up of multiple concentric regions. For different concentric circle regions, the proposed DRF module is learned to generate the adaptive responses that can play the roles of enhanced, suppressive, or irrelevant effects on the feature responses, and hence achieve the effects of adaptive RF (Figure 1B). By modeling DRF mechanisms of BVS, our proposed DRF module can effectively integrate both global and local context information for feature extraction and representation under the premise of a slight increase in parameters and computational cost.

## 2. Related work

We deliberate the related work from three aspects that are dynamic RF, attention mechanism, and transformer in computer vision, respectively. In a biological neural processing mechanism, when the photoreceptors are stimulated, the nerve impulses coding various light information are transmitted to the downstream neurons through the receptive field processing and integration (Chen et al., 2013; Angelucci et al., 2017). The receptive field is generally defined as a specific region of sensory space where a visual stimulus can elicit electronic responses of a neuron in a specific visual area (Kuffler, 1953; Hubel and Wiesel, 1962). The canonical RF mechanism exists in the peripheral sensory neurons, relay nucleus neurons, and neurons in the cerebral cortex sensory area of organisms. However, the nature and size of the receptive fields in different neural processing stages are not consistent, and the RF mechanisms in the same location of different organisms can also differ. For example, the receptive fields of the optic ganglion cells of cats and monkeys are composed of both excitatory and inhibitory fields that form concentric circles (Cavanaugh et al., 2002; Series et al., 2003), while for the receptive fields of optic ganglion cells of frogs, rabbits, pigeons, etc., except for the concentric circles, there are also receptive fields that can only react to special stimulus such as a moving bar with the specific orientation.

The RF imitated in CNN (Simonyan and Zisserman, 2014; Hu et al., 2019) generally shares similar concepts as the biological one but with some key differences. The size of RF in CNN is generally determined by the size of the convolution kernel, the pooling layer, and the depth of the network. Various strategies can be used to increase the size of RF, such as increasing the kernel size, stacking more layers (e.g., increasing the network depth) (Szegedy et al., 2015), implementing sub-sampling, and dilated convolution (Chen et al., 2017). Simply increasing the kernel size or deepening the network depth, although theoretically a larger receptive field can be obtained and more information can be extracted, the number of parameters and calculations are significantly increased either, which will lead to the over-fitting and even performance degradation (Nielsen, 2015). Furthermore, not all receptive fields in CNN are effective and make the same contribution to the output feature responses (Luo et al., 2016; Dai et al., 2017).

Another pipeline to implement the dynamic function of a BVS is adopting the attention mechanism, which also takes inspiration from the early visual information processing in the biological visual system. Attention mechanisms adopted in deep learning can be generally divided into two categories: channel domain and spatial domain. By assigning different weights to various channels or regions in the space, instead of treating spatial locations or all channels as having the same importance when performing convolution or pooling operations in the past, many approaches exploiting attention mechanisms make the network focus on the extraction of more important information. SENet (Hu J. et al., 2018) tried the first attempt to adaptively adjust the feature responses of each channel using the Squeeze-and-Excitation module from the channel-wise level. BAM (Park et al., 2018) and CBAM (Woo et al., 2018) produced a spatial attention module, which introduces the dynamic spatial representation by global pooling. However, this method can only capture local information. Self-attention mechanism transplanted from natural language processing (NLP) (Hu H. et al., 2018; Hu et al., 2019; Zhao et al., 2020; Srinivas et al., 2021) takes all global information into account during one operation can further capture the long-range dependence compared with traditional convolution neural networks. For example, the non-local neural networks (Wang et al., 2018) employ self-attention as an additional block interspersed in the Resnet backbone and Bottleneck transformers (Srinivas et al., 2021) replaces the spatial convolution with multi-head self-attention in the final three bottleneck blocks of Resnet. Both approaches obtain significant performance improvement in object detection. The conventional self-attention mechanism primarily measures the correlation between pixels by calculating the inner product of the feature vector corresponding to a pixel, then generating a weight matrix based on this correlation, and performing a weighted summation of features to capture the dependencies of distant pixels. Although many self-attention-based models tried to reduce the memory and computation cost by reducing the number of channels in the embedding matrix or performing self-attention computations only on low-resolution abstract features, the computational complexity is still huge because one performance requires two times vector inner product calculations for all feature vectors.

Hence, some recent works (Hu et al., 2019; Zhao et al., 2020) tried to design variants of self-attention and just employ self-attention within a patch area with fixed size instead of crossing all the image, which significantly reduces the amount of computation. However, the patch size is an intermediate variable that needs to be designed manually based on experience and cannot be changed adaptively. Contextual transformer (Li et al., 2022) simplifies the production of dynamic attention matrix by abandoning the learning of query–key relationship. Their network captures contextual information using larger kernel convolution and obtains dynamic spatial attention by superimposing two 1 × 1 convolutions. Although this method made up of the shortcoming of the limited receptive field of traditional CNN by acting on a global dynamic spatial attention, it is still inferior in capturing long-distance interaction compared with the original transformer module.

In this study, we simulate the dynamic receptive field (DRF) mechanism of the biological visual system to design a more effective receptive field processing strategy in CNNs, which can reflect the strong adaptability of biological neurons for different inputs. The proposed DRF module can obtain long-range responses encoding useful contexture information through global feature guidance. At the same time, the proposed DRF module also achieves a relative position encoding mechanism, which adaptively assigns different levels of importance to the global feature pixels in different receptive field regions, and hence achieves the purpose of dynamically adjusting the receptive field.

## 3. Proposed method based on DRF mechanisms

**Figure 2:**
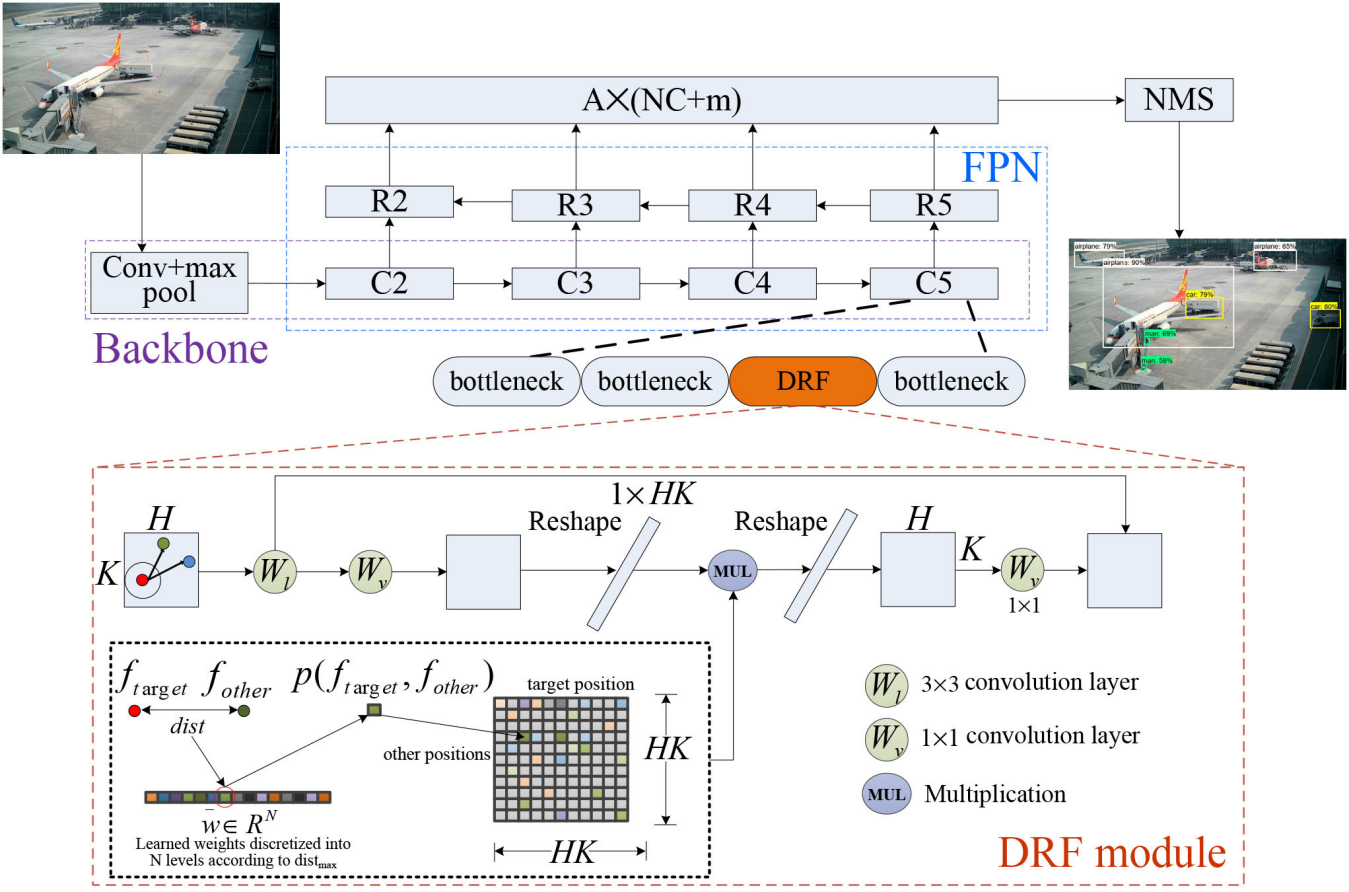
The structure of network built on our proposed DRF module for object detection.

The structure of the proposed network for object detection is shown in Figure 2. Generally, our model builds on the Feature Pyramid Network (FPN) (Lin et al., 2017a), which consists of the backbone, where the proposed DRF module is incorporated into each stage (e.g., C5 is shown in Figure 2) of the backbone for enhancing the capability of feature extraction and representation. We deliberate each component as follows in detail emphasizing the proposed DRF module.

### 3.1. Dynamic receptive field module

The proposed module generally includes the following four core steps: First, for an input feature map processed by DRF, we divide the relative distance corresponding to different receptive field regions into N different discrete levels and assign a weight that is learned by the neural network to each level. Second, for each feature point, we calculate the relative distance between the feature point and its surrounding feature points, divide the calculated relative distance into N different levels, and then assign the corresponding learned weight in step 1 to each level. By this operation, we obtain a weight matrix encoding the distance relation information of all other feature points related to this target feature point. Third, we multiply the weight matrix of the feature point obtained in step 2 by the feature map to obtain the excitation output of this target feature point. For an input feature map, we finally obtain the global guidance response of each feature point based on the relative positional relationship. Fourth, to preserve the local information in the excitation output, the feature map is input to a convolutional layer with a fixed convolution kernel to obtain the local response output of the feature map. Then, we add the local response output of each feature point and its global guidance response obtained in step 3 together to obtain the final output feature. The mathematical details for each step are as follows:

Taking a target feature point *f*(*x*_*target*_, *y*_*target*_) on the feature map *F* as an example, (*x*_*target*_, *y*_*target*_) represents the spatial position of the target feature point. *f*(*x*_*other*_, *y*_*other*_) represents other feature points surrounding the target feature point on the feature map *F*, and (*x*_*other*_, *y*_*other*_) represents the spatial position of other feature points. The relative spatial distance between the target feature point and the surrounding feature points is defined as follows:


(1) $$\begin{array}{l} dist\left(f_{target},f_{other}\right)=\sqrt{{\left(\frac{x_{target}-x_{other}}{H}\right)}^{2}+{\left(\frac{y_{target}-y_{other}}{K}\right)}^{2}} \end{array}$$

*K* and *H*, respectively, represent the length and width of the feature map *F*, and the maximum value of the relative distance calculated according to Equation (1) is $\sqrt{2}$, due to that we calculate the normalized distance. We divide the maximum normalized relative distance into *N* discrete levels and assign each level a weight value ω, which will be learned by the gradient back-propagation algorithm. Hence, different learned weight values form a vector $\bar{\omega }\in R^{N}$.

For the feature point at each spatial position in the feature map *F*, we calculate the relative distance between the target feature point and other surrounding feature points according to Equation (1) and map the calculated relative distance *dist*(*f*_*target*_, *f*_*other*_) into *N* different levels. The weight value assigned to each level is calculated as follows:


(2) $$\begin{array}{l} \begin{array}{c} \rho (f_{target},f_{other})=\omega_{k}\;if\;\frac{\left(k\cdot \sqrt{2}\right)}{N}\leq dist(f_{target},f_{other})\\ \;<\frac{\left((k+1)\cdot \sqrt{2}\right)}{N},\;k\in [0,1,\cdots \;,N] \end{array} \end{array}$$

According to Equation (2), we assign the weight value ω_*k*_ in the corresponding weight vector $\bar{\omega }\in R^{N}$ to all feature points on this level, so as to obtain a weight coefficient matrix *P*∈*R*^*HK*×*HK*^ of each feature point on the entire feature map relative to its surrounding feature points. Several examples of visualized weight maps learned from the dataset are shown in Figure 1C. Finally, the weight coefficient matrix is normalized point-to-point by a Softmax function as follows to obtain a weight coefficient matrix *S*∈*R*^*HK*×*HK*^ within the values of [0, 1].


(3) $$\begin{array}{l} s(f_{target},f_{other})=\frac{exp(\rho (f_{target},f_{other}))}{\sum_{other}exp(\rho (f_{target},f_{other}))} \end{array}$$

For an input feature map *F*^*C*×*H*×*K*^, where *C* denotes the number of channels, we first pass the feature map into a convolutional layer *W*_*v*_ with the size of 1 × 1.


(4) $$\begin{array}{l} V=F⊗W_{v} \end{array}$$

Then, for each feature point in the feature map, we calculated the product of the feature map *V* and the weight coefficient matrix *S*, so as to obtain the excitation output of the feature point after the weighted average of the calculated feature map *V* and the weight coefficient matrix *S*.


(5) $$\begin{array}{l} o=S^{T}V \end{array}$$

We perform the operations of the above Equations (1)–(5) on each feature point in the feature map *F*^*C*×*H*×*K*^, and finally obtain the output of the entire feature map *O*^*C*×*H*×*K*^. Finally, *O*^*C*×*H*×*K*^ is further passed into a 1 × 1 convolutional layer of *W*_*v*_ to get the global response output of the feature map *G* = *O*⊗*W*_*v*_.

To further retain the local context information, we pass the input feature map *F*^*C*×*H*×*K*^ into a convolutional layer *W*_*l*_, with a fixed convolution kernel with the size of 3 × 3 to obtain the local response *L* = *F*⊗*W*_*l*_. The final output of the feature map after being processed by the convolutional neural network module based on the biological visual dynamic receptive field mechanism described in this study is the addition of the global response output *G* with the local response *L*. Figure 2 shows the details of biologically inspired DRF module. In practical computation, to reduce computation and memory consumption of the proposed DRF module, first, we designed our DRF model as a bottleneck-like architecture by reducing the number of feature channels through a convolution with kernel size 1, and then feeding the output into the DRF module and restoring the number of feature channels through another convolutional layer.

### 3.2. Multi-head DRF

**Figure 3:**
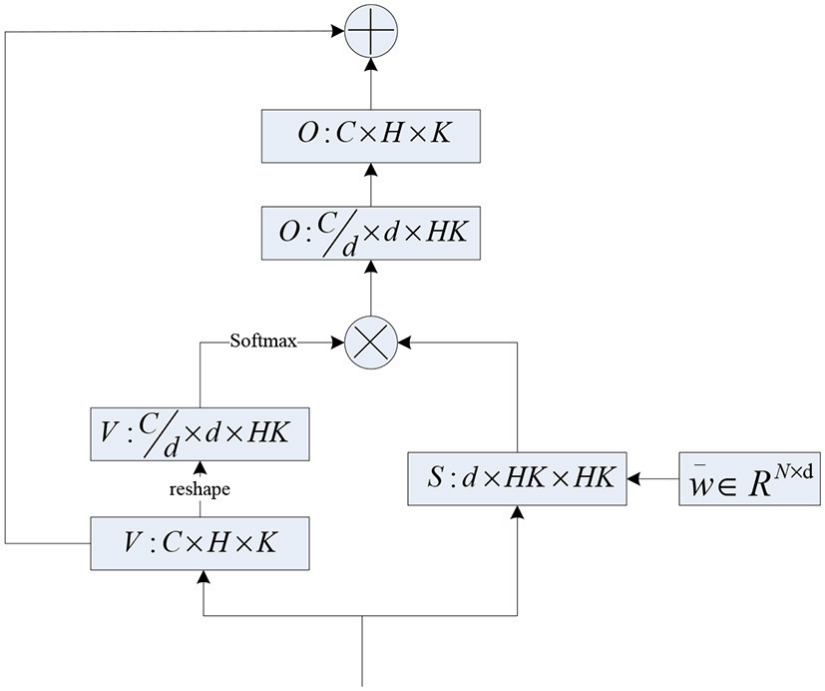
The computation flowchart of proposed multi-head DRF.

Inspired by multi-head self-attention, we also devised a multi-head DRF module as shown in Figure 3. Concretely, we increased the learned weight vector $\bar{\omega }\in R^{N}$ by one more dimension $\bar{\omega }\in R^{N\times d}$, and *d* indicates the head-dim. Hence, we get the multi-head weight coefficient matrix *S*∈*R*^*d*×*HK*×*HK*^ and the multi-head feature map $V^{\frac{C}{d}\times d\times HK}$. The final results of multi-head DRF are obtained by multiplying *S*∈*R*^*d*×*HK*×*HK*^ with $V^{\frac{C}{d}\times d\times HK}$ using the regular matrix manipulation as shown in Figure 3. The proposed multi-head design allows the DRF module to extract feature information from multiple subspaces and facilitate the representation capacity of features. The total computational cost of the multi-head DRF is similar to that of a single-head DRF.

### 3.3. The object detection network

As shown in Figure 2, we adopted the Feature Pyramid Network (FPN) as the detection network and use FPN levels from 2 to 5 for feature extraction. We transformed the output of FPN levels *C*_2_, *C*_3_, *C*_4_, and *C*_4_ to the new feature maps *R*_2_, *R*_3_, *R*_4_, and *R*_5_ using one convolutional layer with kernel size 1. The feature maps *R*_3_, *R*_4_, and *R*_5_ are upsampled using the nearest neighbor by a factor of 2, and then the upsampled *R*_3_, *R*_4_, and *R*_4_ are merged with the corresponding original feature maps *C*_3_, *C*_4_, and *C*_4_ through element-wise addition. Finally, we feed the summation result into another 1 × 1 convolutional layer to get the final detection results. To reduce the number of parameters, we set the group number as 8 for each convolutional layer.

Experiments show that the grouped convolution does not reduce the final detection effect while reducing the number of parameters sufficiently. This process is described by Figure 2. The output of each feature map point is a one-dimensional vector whose dimension is *A*×(*NC*+*m*), A represents the number of anchors, *NC* refers to the number of categories of the training data, and *m* indicates the number of confidence value and the transformation of the width and height relative to the corresponding anchor. To evaluate the performance of the proposed DRF module, we incorporate the DRF module into each stage of the backbone as shown in Figure 2, where the structure of a specific module is amplified for better visualization.

### 3.4. Loss function

The loss function used in our model includes three parts as follows:

(1) We adopt the conventional Binary Cross Entropy (BCE) as our first classification loss function. When calculating the classification loss function, only the loss of positive samples is considered. The positive samples are determined by the proposed anchor matching strategy, which will be described later in detail. In the following formula, *y* is the classification output vector, and *y*′ is the target one-hot label, which has been smoothed (Müller et al., 2019).


(6) $$\begin{array}{l} L_{class}=BCE_{Loss}\left(y,y^{\prime }\right) \end{array}$$

(2) Focal Loss (Lin et al., 2017b) was usually designed to down-weight easy examples in object detection, and thus focus training on hard negatives. Hence, we also use the focal loss to deal with the imbalance of difficult and easy samples.


(7) $$\begin{array}{l} L_{conf}=FL\left(p,p^{\prime }\right)=-{\left(1-abs\left(p-p^{\prime }\right)\right)}^{\gamma }\times BCE_{Loss}\left(y,y^{\prime }\right) \end{array}$$

Where *p* is the confidence output, and *p*′ is the target confidence output. We set the confidence value to 1.0 corresponding to the positive sample, and 0.0 corresponding to the negative sample. The focal factor γ was set as 2.0 in our experiment to adjust the importance of easy/hard examples.

(3) Generally, L1 or L2 norm is used to regress the four coordinate points of the predicted box and use IOU (Intersection over Union) to evaluate the accuracy of the prediction. However, both measures are not completely positively correlated. Many variants of IOU loss have been proposed (Yu et al., 2016; Rezatofighi et al., 2019; Zheng et al., 2020) to address this issue. Here, we use CIOU loss (Zheng et al., 2020) as the final regression loss function.


(8) $$\begin{array}{l} L_{reg}=L_{CIOU}\left(b,b^{gt}\right) \end{array}$$

Where *b* and *b*^*gt*^, respectively, indicates the predicted coordinates of the box and the corresponding ground truth. In summary, our loss function can be expressed as following.


(9) $$\begin{array}{l} LOSS=1^{obj}L_{class}+\left(1^{obj}\;\&\;1^{noobj}\right)L_{conf}+1^{obj}L_{reg} \end{array}$$

1^*obj*^ is the positive sample mask and 1^*noobj*^ is the negative sample mask, which is determined by our matching strategy.

### 3.5. Proposed matching strategy

We benchmarked the max-IOU assigner strategy with some improvements. First, for each ground truth, we calculate the IOU with all anchors and select the top N with the largest IOU as the candidates. Then, referring to ATSS (Zhang et al., 2020), we calculate the L2 distance between the center points of the candidate anchors and the center point of the ground truth and select the first k anchors with the smallest distance as the further candidates. Finally, to make the anchors match the ground truth more, we remove the anchors whose center point is located outside the ground truth box. In particular, when the same anchor is accidentally selected by multiple ground truths, we assign it to the ground truth with the largest IOU.

In addition, we found through experiments that in a few cases, some ground truth cannot match any anchors, which is extremely unfavorable for target detection. To avoid such a situation, we additionally take out the anchor, which has the largest IOU with each ground truth and make it responsible for predicting the position offset. In this way, ground truth can match up to k anchors. In our experiment, we set N to 10 and k to 5 to achieve a relatively good test effect. The proposed matching strategy process is shown in Algorithm 1.

**Algorithm 1:** Proposed matching strategy

1: Calculate IOU between all the ground truths and anchors
2: **for** each ground truth *g*_*i*_ **do**
3: Select top N anchors with largest IOU as candidate set Θ_*i*_
4: In Θ_*i*_, select first *k* anchors with smallest *L*_2_ distance between candidate anchors and *g*_*i*_ as further candidate set Ω_*i*_
5: In Ω_*i*_, remove anchors whose center point is located outside the *g*_*i*_ box and get candidate anchor set Δ_*i*_
6: **end for**
7: **for** each anchor α_*k*_ **do**
8: If α_*k*_∈Δ_*i*_∪Δ_*j*_ (Δ_*j*_ is the candidate anchor set for *g*_*j*_), remove α_*k*_ from Δ_*i*_ if the IOU between α_*k*_ and *g*_*i*_ is smaller than IOU between α_*k*_ and *g*_*j*_
9: **end for**
10: **for** each ground truth *g*_*i*_ **do**
11: Get the anchor with the largest IOU, assign it to *g*_*i*_, and put it into the collection Δ_*i*_
12: **end for**
13: Output: the candidate anchor set Δ_*i*_, *i* = 1, 2… for each ground truth *g*_*i*_, *i* = 1, 2…

**Figure 4:**
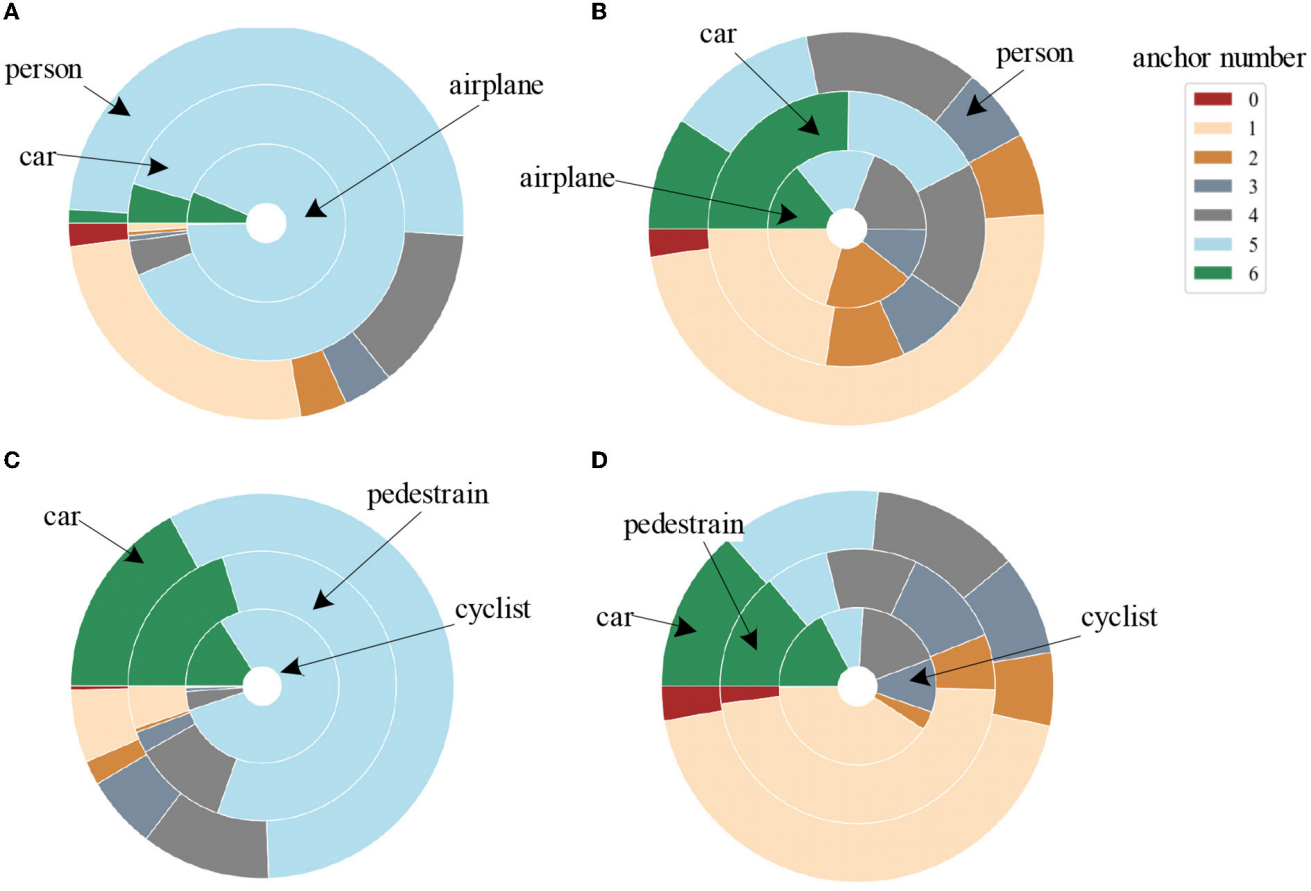
Comparison of the number of matched anchors for different categories between our proposed matching strategy [**(A)** for the airport dataset, **(C)** for the KITTI dataset] and the commonly used max-IOU matching strategy [**(B)** for the airport dataset, **(D)** for the KITTI dataset]. Different concentric circles represent various categories of ground truth, and different colors indicate the number of anchors matched by each ground truth.

Figure 4 shows the comparison of the number of anchors matches between our proposed matching strategy (Figures 4A, C) and the common max-IOU matching strategy (Ren et al., 2015; Liu et al., 2016) (Figures 4B, D) on two benchmark datasets. It can be seen that the number of anchors matched by our method is more balanced. The red area indicates that the ground truth does not match any anchors at all. This part has a huge impact on the detection. Hence, the matching strategy needs to avoid the situation where the ground truth cannot completely match the anchor as much as possible. Moreover, the closer the distance between the ground truth and the matching anchor, the greater the intersection of IOU, and the better and faster return of the target location information by matching the anchor during training. Subsequent experiments are shown in Table 5 also show that our proposed matching strategy significantly improves the detection results of small targets compared with the traditional IOU-max method (Ren et al., 2015; Liu et al., 2016).

## 4. Experimental settings and results

**Figure 5:**
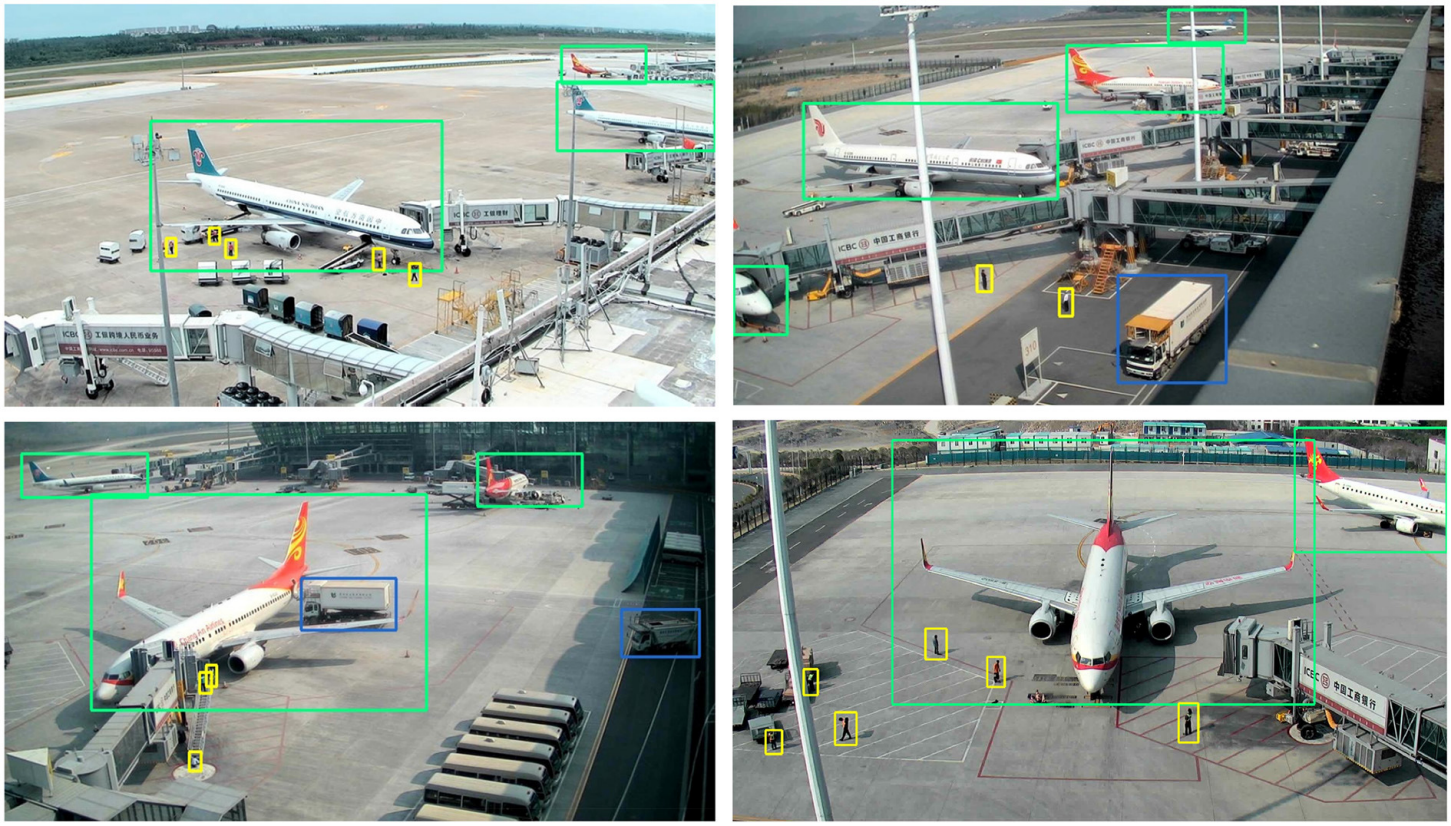
Several examples of detection results on the airport dataset. We can see that the objects with very small size such as pedestrians have been clearly detected by our proposed model.

We test the proposed model on four datasets with tasks for object detection and image recognition. Concretely, we conduct experiments on our airport scene dataset and the public benchmark KITTI dataset (Geiger et al., 2012) to verify the benefits of the proposed DRF/M-DRF for object detection. We also report results using CIFAR-10 and CIFAR-100 datasets (Krizhevsky and Hinton, 2009) for image recognition to prove that our proposed DRF/M-DRF module can effectively extract the target information for representation. The airport scene dataset containing 5,549 images is extracted from the public surveillance videos of the civil airport scene and the videos shoot by dozens of camera positions distributed around the airport terminals in China. The biggest challenge on this dataset is the small target detection of the human category shown in Figure 5, which occupies a quite small area in the image. The average value of the size of the human target is only 1,276 pixels and the smallest target occupies only 9 pixels. It is easy to lose the characteristic information of the small target during the down-sampling process of the neural network, and hence resulted in false and missed detection.

The KITTI dataset (Geiger et al., 2012) is currently the largest dataset for computer vision algorithm evaluation in autonomous driving scenarios. KITTI contains images collected from different scenes with various degrees of occlusion and truncation. There are a total of 7,481 fully annotated images. We simplify the categories of the KITTI dataset to three categories, which are ‘car', ‘pedestrian', and ‘cyclist' at different scales for detection. Among them, the categories of ‘pedestrian' and ‘cyclist' are obviously small targets. For the image classification task, we used CIFAR-10 and CIFAR-100 (Krizhevsky and Hinton, 2009) consisting of 120,000 colored natural scene images with the size of 32 × 32 pixels to verify the feature extraction ability of our module on the quite low-resolution image.

### 4.1. Experimental details

Our experimental hardware platform is NVIDIA TI 2080 GPU and Intel I7-9700 CPU with Cuda 11.1 and Cudnn 7.65. Our network is based on pytorch 1.4.0, and the weight of the convolutional layers adopts a normal distribution initialization with a mean value of 0.0 and a standard deviation of 0.1, all bias is set to be a constant of 0.0. The weight of the regularized BatchNorm (Ioffe and Szegedy, 2015; Santurkar et al., 2018) layers is designed to be constant 1.0, and the deviation is 0.0.

### 4.2. Experiments on target detection

In this section, we build a target detection framework to verify and analyze the effectiveness of our proposed DRF/M-DRF module. The complete network architecture is shown in Figure 2. The FPN structure and loss design have been detailed in Sections 3.3, 3.4, respectively. We use the SGD optimizer with momentum of 0.9 and weight decay of 0.0005 to train our network, and the learning rate decay strategy uses a cosine decay scheduler (Takahashi et al., 2019), with a warmup of two epochs in the beginning (Goyal et al., 2017). Specifically, our total training epoch number is 30, the initial learning rate is set as 0.01, and the end learning rate is 0.0001.

#### 4.2.1. Experiments with a single M-DRF block

Tables 1–3 exhibit the detection results on both the airport dataset and the KITTI dataset by replacing a single original residual block of ResNet50 and ResNeXt50 backbone with our proposed M-DRF block. The replacement location is right before the last residual block of each stage. Experimental results clearly showed that exploiting a single M-DRF module can effectively improve the performance of target detection on two datasets. As shown in Tables 1, 2, the test results on different backbones show that adding our module to any stages of the backbone can produce a significant improvement. When adding a high-resolution stage (e.g., res2 and res3), the improvement effect is weaker, but the increase in the number of parameters is more. Therefore, we suggest that the replacement position of the DRF module should be in the last two stages (e.g., res4 and res5) to obtain a balance between the performance and the number of parameters. In our subsequent experiments, the DRF insertion position is also placed in the latter two stages. In addition, by comparing the improvement effect of each category for detection, we can observe that the performance boost of our method is particularly obvious for small object detection, such as on categories of vehicles, persons, cyclists, and pedestrians. This means that our DRF module can indeed improve the feature expression and integration capabilities of backbones.

**Table 1:** Detection results on airport dataset by replacing the penultimate residual block of the different stages of Resnet50 backbone with the proposed M-DRF (*M* = 8).

**ResNet50, image size 320 × 320**	**Airplane**	**Car**	**Person**	**mAP ↑**
Baseline	98.8	95.1	55.1	83.0
res2	98.96 (+0.16)	95.43 (+0.33)	53.74 (–1.36)	82.71 (–0.29)
res3	99.14 (+0.34)	95.60 (+0.50)	56.25 (+1.15)	83.66 (+0.66)
res4	99.42 (+0.62)	96.27 (+1.17)	55.98 (+0.88)	83.89 (+0.89)
res5	99.24 (+0.44)	95.64 (+0.54)	58.26 (+3.15)	84.38 (+1.38)

The numbers in parentheses indicate the improvement of our M-DRF over the baseline. The symbol ↑ indicates that the higher the mAP number, the better the performance.

**Table 2:** Detection results on airport dataset by replacing the penultimate residual block of the different stages of ResneXt50 backbone with the proposed M-DRF (*M* = 8).

**ResNeXt50, image size 320 × 320**	**Airplane**	**Car**	**Person**	**mAP ↑**
Baseline	98.87	95.34	55.66	83.29
res2	99.17 (+0.3)	95.77 (+0.43)	56.30 (+0.64)	83.75 (+0.46)
res3	99.26 (+0.39)	95.49 (+0.15)	57.66 (+2.0)	84.14 (+0.81)
res4	99.41 (+0.54)	95.53 (+0.19)	56.79 (+1.13)	83.91 (+0.62)
res5	99.11 (+0.24)	95.37 (+0.03)	55.69 (+0.03)	83.39 (+0.1)

The numbers in parentheses indicate the improvement of our M-DRF over the baseline. The symbol ↑ indicates that the higher the mAP number, the better the performance.

**Table 3:** Detection results on KITTI dataset by replacing the penultimate residual block of the last stage of Resnet50 and ResneXt50 backbone with the proposed M-DRF (*M* = 8).

**KITTI, image size 320 × 320**	**Car**	**Pedestrian**	**Cyclist**	**mAP ↑**
ResNet50	93.91	62.65	63.66	73.40
ResNet50 + DRF (res4)	94.22 (+0.31)	63.78 (+0.12)	66.61 (+2.95)	74.87 (+1.47)
ResNet50 + DRF (res5)	94.29 (+0.38)	63.23 (+0.58)	68.68 (+5.02)	75.40 (+2)
ResNeXt50	94.78	66.41	70.65	77.28
ResNeXt50 + DRF (res4)	94.80 (+0.02)	65.96 (–0.45)	71.60 (+0.95)	77.45 (+0.17)
ResNeXt50 + DRF (res5)	94.99 (+0.21)	67.60 (+1.19)	70.60 (–0.05)	77.73 (+0.45)

The numbers in parentheses indicate the improvement of our M-DRF over the baseline. The symbol ↑ indicates that the higher the mAP number, the better the performance.

#### 4.2.2. Experiments with head-num of M-DRF block

We further investigate how the head-num of adopted multi-head-DRF affects the detection performance on the airport and KITTI datasets. We use ResNet50 as a baseline and replace the penultimate bottleneck with M-DRF block with a different head-num. Table 4 indicates that the detection results show an upward trend with the increase of numbers of exploited head-num of M-DRF block, which clearly proved the effectiveness of the proposed multi-head design on facilitating the capacity of extracting feature information with a slight increasing in parameter compared with a single-head DRF. Compared with the single-head DRF module, the multi-head mechanism allows different heads to pay attention to different receptive field areas and realizes multiple independent attention calculations, meaning allows the DRF module to extract feature information from multiple subspaces, which can further improve the expression ability of the model.

**Table 4:** Detection results on KITTI dataset and Airport dataset by replacing the penultimate residual block of the last stage of Resnet50 with the proposed head-num of M-DRF block.

**Image size 320 × 320**	**KITTI dataset**				**Airport dataset**			
	**Car**	**Pedestrian**	**Cyclist**	**mAP** ↑	**Car**	**Pedestrian**	**Person**	**mAP** ↑
ResNet50, basline	93.91	62.65	63.66	73.40	98.8	95.1	55.1	83.0
ResNet50 + DRF (*M* = 1)	94.14	62.75	66.22	74.37	99.22	95.3	55.53	83.35
ResNet50 + DRF (*M* = 2)	94.22	63.80	67.91	75.31	99.31	95.27	54.86	83.15
ResNet50 + DRF (*M* = 4)	94.26	64.87	68.52	75.88	99.31	95.14	53.93	82.79
ResNet50 + DRF (*M* = 8)	94.09	63.74	66.69	74.84	99.24	95.64	58.26	**84.38**
ResNet50 + DRF (*M* = 16)	94.10	64.79	68.91	**75.93**	99.29	95.22	55.03	83.19

The symbol ↑ indicates that the higher the mAP number, the better the performance.

#### 4.2.3. Effects of proposed matching strategy

We further conduct experiments using different backbones on the two datasets to verify the effectiveness of the proposed matching strategy. As shown in Section 3.5, the proposed matching strategy can make the number of anchors matched by the ground truth more balanced and effectively avoid small targets being missed. Experimental results shown in Table 5 further demonstrate that the proposed matching strategy significantly improves the detection results compared with using the traditional max-IOU method (Ren et al., 2015; Liu et al., 2016), especially for small target detection, such as categories of person, pedestrian, and cyclist.

**Table 5:** Detection results on KITTI dataset and Airport dataset using two different matching strategies for comparison.

**Image size 320 × 320**	**Airport dataset**				**KITTI dataset**			
	**Airplane**	**Car**	**Person**	**mAP** ↑	**Car**	**pedestrian**	**Cyclist**	**mAP** ↑
ResNet50 + iou-max	99.40	95.08	51.5256	82.0	87.57	50.14	53.39	63.7
ResNet50 + our strategy	98.8	95.1	**55.1**	**83.0**	93.91	**62.65**	**63.66**	**73.40**
ResNeXt50 + iou-max	99.29	95.30	53.48	82.69	88.59	53.95	56.91	66.48
ResNeXt50 + our strategy	98.87	95.34	**55.66**	**83.29**	94.78	**66.41**	**70.65**	**77.28**

The symbol ↑ indicates that the higher the mAP number, the better the performance.

### 4.3. General detection results compared with SOTAs

We quantitatively compare our method with several SOTA methods including SSD (Liu et al., 2016), Faster RCNN (Ren et al., 2015), RetinaNet (Lin et al., 2017b), YOLO v3 (Redmon and Farhadi, 2018), and YOLO v5 (Jocher et al., 2020). The main information involved in the comparison includes the input size of image, the size of parameters, the flops of the model, and the test results measured with mAP for each class of the experimental datasets. For a fair comparison with SOTA backbones, we also report the results of replacing only the backbone on our detector framework alone. Specifically, we compared the classic Resnet50, Resnet101 and ResneXt50, and ResneXt101 backbones of different depths. Among these, for the Resnet backbone, we directly replace the bottleneck with our proposed M-DRF. For the ResneXt backbone, we replace the 3 × 3 convolution in M-DRF for generating the local response with the same group convolution as in ResneXt.

**Figure 6:**
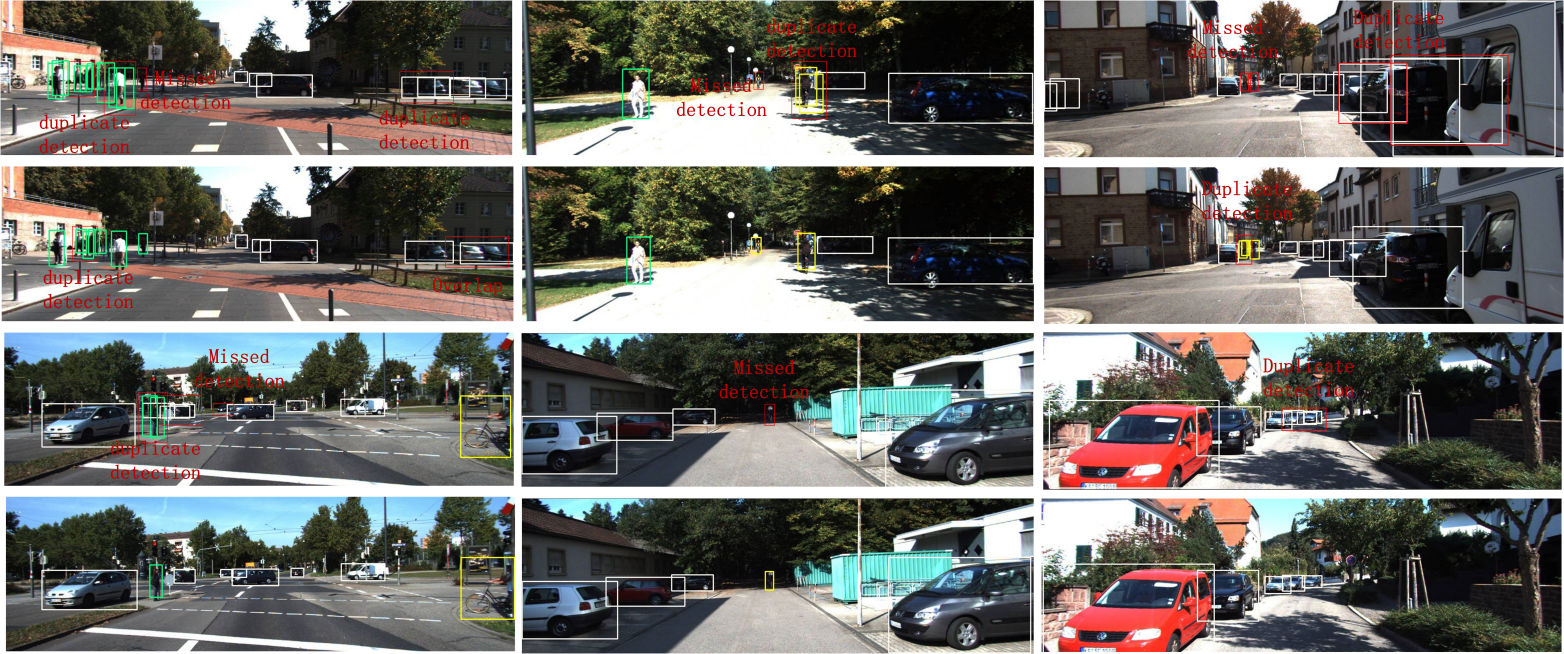
Several examples of detection results on the KITTI dataset with and without the proposed DRF using the FPN and the proposed matching strategy as the object detection framework. We can see that the objects with very small size such as pedestrians in the middle image have been clearly detected (the second and fourth rows) compared with that of the detection results without DRF (the first and third rows).

For Resnet50 and ResneXt50, the replacement position of M-DRF is the penultimate residual module of the last stage. For Resnet101 and ResneXt101, we replaced three residual modules, which are the 5th, 11th, and 17th residual modules of stage3, respectively. Tables 6, 7 show that our proposed approach outperforms several recent SOTA approaches on two benchmark datasets under the same or even lower parameters and flops. Figures 5, 6, respectively, show several example detection results on the two datasets. For experimental comparison in Figures 5, 6, we use the proposed object detection framework that is generally similar to Yolo v3 but with some key differences. Concretely, we adopt the Feature Pyramid Network (FPN) as the feature extraction network and use FPN levels from 2 to 5 for feature extraction. We use Renet50 as the backbone and incorporate the proposed DRF into the backbone for experimental comparison. Furthermore, we also adopt the different loss function and matching strategy as shown in previous sections compared with the original Yolo v3. Our proposed model incorporated into the DRF module is very effective in detecting small targets compared with the one without the DRF module in which many small and difficult targets are missed.

**Table 6:** Performance comparisons with various SOTA object detection frameworks on the airport scene dataset.

**Method**	**Backbone**	**Size**	**Para(MB)**	**Flops(GB)**	**Airport dataset**			
					**Airplane**	**Car**	**Person**	**mAP**↑
SSD	Vgg16	300*300	22.9	30.59	90.74	80.96	23.79	65.17
		512*512		88.01	90.78	88.63	38.42	72.61
retinaNet	Resnet18	600*600	18.89	18.48	94.49	76.91	25.02	65.48
	Resnet50		34.69	36.72	93.62	77.36	21.25	64.08
Faster Rcnn	Vgg16	~	130.39	~	90.8	79.9	29.5	66.7
	Resnet50		26.98		90.8	80.5	26.2	65.8
YOLO v3	Darknet53	416*416	60.09	65.5	98.0	84.2	35.3	72.5
		640*640		155.1	99.1	92.4	42.9	78.1
YOLO v5	YOLOv5s	320*320	6.69	15.8	98.5	84.7	38.4	73.9
	YOLOv5m	320*320	19.89	47.9	99.2	87.9	41.4	76.1
	YOLOv5l	320*320	43.98	107.7	99.2	90.0	43.9	77.7
	YOLOv5x	320*320	82.19	203.8	99.3	90.9	45.2	78.4
Classical Backbone	Resnet18	320*320	47.304	14.32	98.52	89.77	36.32	74.87
	Resnet50	320*320	22.58	7.99	98.8	95.1	55.1	83.0
	RXt50(32 × 4*d*)	320*320	22.07	8.27	98.87	95.34	55.66	83.29
	SE-Resnet50	320*320	26.189	8.59	99.26	95.56	53.32	82.71
	Resnet101	320*320	40.82	15.11	99.10	95.48	55.44	83.34
	RXt101(32 × 8*d*)	320*320	82.88	31.49	99.23	94.91	53.88	82.67
DRF Backbone	DRF-Res50	320*320	23.08	8.04	**99.42**	**96.27**	**55.98**	**83.89**
	DRF- Res101	320*320	41.07	15.21	**99.38**	**96.11**	**57.86**	**84.45**
	DRF-RXt50(32 × 4*d*)	320*320	8.47	24.08	**99.41**	**95.53**	**56.79**	**83.91**
	DRF-RXt101(32 × 8*d*)	320*320	88.89	33.84	**99.28**	**95.41**	**54.97**	**83.22**

The symbol ↑ indicates that the higher the mAP number, the better the performance.

**Table 7:** Performance comparisons with various SOTA detection frameworks on the KITTI dataset.

**Method**	**Backbone**	**Size**	**KITTI dataset**			
			**Airplane**	**Car**	**Person**	**mAP↑**
SSD	Vgg16	300*300	81.04	37.10	40.66	52.93
		512*512	86.42	42.00	44.25	57.56
retinaNet	Resnet18	600*600	91.46	78.96	79.67	83.36
YOLO v3	Darknet53	416*416	91.4	67.8	70.6	76.6
		640*640	94.8	77.3	81.3	84.5
Classical backbone	Resnet50	320*320	93.91	62.65	63.66	73.40
	Resnet101	320*320	95.53	67.72	76.68	79.98
	ResneXt50(32 × 4*d*)	320*320	94.78	66.41	70.65	77.28
	ResneXt101(32 × 8*d*)	320*320	96.20	73.45	81.60	83.75
DRF+Backbone	DRF-Resnet50	320*320	**94.22**	**63.78**	**66.61**	**74.87**
	DRF-Resnet101	320*320	**95.93**	**70.38**	**78.3**	**81.54**
	DRF-ResneXt50(32 × 4*d*)	320*320	**94.80**	**65.96**	**71.60**	**77.45**
	DRF-ResneXt101(32 × 8*d*)	320*320	**96.55**	**75.51**	**83.42**	**85.16**

The symbol ↑ indicates that the higher the mAP number, the better the performance.

We also design an experiment to compare the deformable convolutional networks (DCN) (Dai et al., 2017; Zhu et al., 2019) with the proposed DRF. DCN achieves the deformable receptive field by adding a two-dimensional position offset to each sampling point position of the standard convolution kernel, and learning this offset from the dataset. DCN enables the trained convolution kernel with variable shapes compared with the standard convolution kernel with a fixed shape. However, DCN is still a local operation because it only exploits the local information of feature points offsetting with the target feature point. As a comparison, the proposed DRF learns different weight values for different receptive field positions in a global scope, and the learned receptive field is larger and can be adjusted dynamically. We can see from Table 8 that our proposed method with DRF clearly performs better than both DCN v1 (Dai et al., 2017) and DCN v2 (Zhu et al., 2019), especially for small targets such as a person on the airport dataset.

**Table 8:** Detection results on Airport dataset by replacing the penultimate residual block of the last stage of Resnet50 with the proposed DRF, DCN v1 (Dai et al., 2017), and DCN v2 (Zhu et al., 2019) for comparison.

**Image size 320 × 320**	**Airport dataset**			
**Method**	**Airplane**	**Car**	**Person**	**mAP** ↑
Resnet50	98.8	95.1	55.1	83.0
Resnet50 + DCN v1	98.83	95.09	53.72	82.54
Resnet50 + DCN v2	99.00	95.41	54.64	83.02
Resnet50 + DRF	99.24 (+0.24)	95.64 (+0.23)	58.26 (+3.62)	84.38 (+1.36)

The symbol ↑ indicates that the higher the mAP number, the better the performance.

### 4.4. Image recognition results on CIFAR

We finally evaluate the proposed DRF on CIFAR-10 and CIFAR-100 datasets for image recognition. The classification network has an architecture consisting of a single convolutional layer, followed by three stages, which have three residual blocks for each stage. We conduct experiments using ResNeXt-29(16 × 32*d*), ResNeXt-29(16 × 16*d*), SKNet, SE-ResNeXt-29(16 × 16*d*),ResNeXt-29(16 × 16*d*)+DRF, and SE-ResNeXt-29(16 × 16*d*)+DRF with last three residual blocks that are replaced by the proposed M-DRF module for comparison. We train our models for 100 epochs in total, using Adam with a mini-batch size of 128 and a weight decay of 1e-5. The initial learning rate is set to 0.001 and decreased by a factor of 10 every 30 epochs. All experiments shown in Table 9 demonstrate that our proposed network building on the DRF can also significantly improve the recognition performance, which proves that our network has a strong ability to extract target features and good portability to be integrated into various tasks (e.g., object detection and image recognition).

**Table 9:** Top-1 accuracy and Top-5 accuracy on CIFAR-10 and CIFAR-100 of the proposed DRF-based network when being evaluated on an image recognition task.

**Models**	**Parameters**	**Flops**	**CIFAR-10**		**CIFAR-100**	
			**Top-1**↑	**Top-5**↑	**Top-1**↑	**Top-5**↑
ResNeXt-29,16 × 32*d*	23.97M	4.05G	88.51	99.58	74.56	93.53
ResNeXt-29,16 × 16*d*	4.92M	826.94M	88.47	99.56	74.16	93.19
SKNet-29	7.19M	874.91M	88.86	99.36	75.23	93.7
ResNeXt-29+DRF,16 × 16*d*	6.33M	881.76M	89.26	**99.74**	**75.27**	**93.92**
SE-ResNeXt-29,16 × 16*d*	5.32M	826.85M	89.63	99.62	73.67	92.84
SE-ResNeXt-29+DRF,16 × 16*d*	6.83M	883.57M	**89.94**	99.68	73.9	92.77

The symbol ↑ indicates that the higher the mAP number, the better the performance.

## 5. Conclusion

We propose a new feature extraction module based on the dynamic receptive field mechanism of visual neurons. Comprehensive experiments on four datasets show that our proposed method can effectively improve the performance of target detection and image recognition compared with other SOTA methods, especially in small target recognition tasks with low resolution. The experimental results support that the neuron's dynamic receptive field mechanism can effectively capture local and global contextual relations, thereby helping the network to detect difficult targets with occlusion and low resolution. Our future work intends to simulate and integrate more visual neuron information processing mechanisms to build a neural network that is more in line with the target detection and recognition mechanism of our visual system, which has become an important driver of progress for next-generation artificial intelligence (Zador et al., 2022).

## Data availability statement

The original contributions presented in the study are included in the article/supplementary material, further inquiries can be directed to the corresponding author.

## Author contributions

MT performed the research. MT, XY, and SH wrote the first draft of the manuscript. MT and BL are responsible for the collection and labeling of the airport dataset. XY is responsible for text verification. MT and SH edited the final manuscript. SH acquired funding for research. All authors contributed to the article and approved the submitted version.
